# Gastrointestinal Manifestations of SARS-CoV-2: Transmission, Pathogenesis, Immunomodulation, Microflora Dysbiosis, and Clinical Implications

**DOI:** 10.3390/v15061231

**Published:** 2023-05-24

**Authors:** Siva Sundara Kumar Durairajan, Abhay Kumar Singh, Udhaya Bharathy Saravanan, Mayurikaa Namachivayam, Moorthi Radhakrishnan, Jian-Dong Huang, Rahul Dhodapkar, Hongjie Zhang

**Affiliations:** 1 Department of Microbiology, School of Life Sciences, Central University of Tamil Nadu, Tiruvarur 610005, India; 2 Department of Biochemistry, School of Biomedical Sciences, Li Ka Shing Faculty of Medicine, The University of Hong Kong, 21 Sassoon Road, Pokfulam, Hong Kong 999077, China; 3 CAS Key Laboratory of Quantitative Engineering Biology, Shenzhen Institute of Synthetic Biology, Shenzhen Institutes of Advanced Technology, Chinese Academy of Sciences, Shenzhen 518055, China; 4 Department of Microbiology, Jawaharlal Institute of Postgraduate Medical Education & Research (JIPMER), Government of India, Puducherry 605006, India; 5 School of Chinese Medicine, Hong Kong Baptist University, Kowloon Tong, Hong Kong 999077, China

**Keywords:** SARS-CoV-2, COVID-19, gastroenteritis, inflammatory bowel disease, pathogenesis, angiotensin-converting enzyme 2, immune responses, gut microflora, therapeutics

## Abstract

The clinical manifestation of COVID-19, caused by the severe acute respiratory syndrome coronavirus 2 (SARS-CoV-2), in the respiratory system of humans is widely recognized. There is increasing evidence suggesting that SARS-CoV-2 possesses the capability to invade the gastrointestinal (GI) system, leading to the manifestation of symptoms such as vomiting, diarrhea, abdominal pain, and GI lesions. These symptoms subsequently contribute to the development of gastroenteritis and inflammatory bowel disease (IBD). Nevertheless, the pathophysiological mechanisms linking these GI symptoms to SARS-CoV-2 infection remain unelucidated. During infection, SARS-CoV-2 binds to angiotensin-converting enzyme 2 and other host proteases in the GI tract during the infection, possibly causing GI symptoms by damaging the intestinal barrier and stimulating inflammatory factor production, respectively. The symptoms of COVID-19-induced GI infection and IBD include intestinal inflammation, mucosal hyperpermeability, bacterial overgrowth, dysbiosis, and changes in blood and fecal metabolomics. Deciphering the pathogenesis of COVID-19 and understanding its exacerbation may provide insights into disease prognosis and pave the way for the discovery of potential novel targets for disease prevention or treatment. Besides the usual transmission routes, SARS-CoV-2 can also be transmitted via the feces of an infected person. Hence, it is crucial to implement preventive and control measures in order to mitigate the fecal-to-oral transmission of SARS-CoV-2. Within this context, the identification and diagnosis of GI tract symptoms during these infections assume significance as they facilitate early detection of the disease and the development of targeted therapeutics. The present review discusses the receptors, pathogenesis, and transmission of SARS-CoV-2, with a particular focus on the induction of gut immune responses, the influence of gut microbes, and potential therapeutic targets against COVID-19-induced GI infection and IBD.

## 1. Introduction

SARS-CoV-2 is a virus that falls under the Nidovirales order, which is known for its positive single-stranded RNA genome [1]. SARS-CoV-1 and SARS-CoV-2 are well-known respiratory illnesses-causing agents. However, they can also infect other organs, including the nasal mucosa, lungs, small intestine, colon, liver, spleen, and brain [2].

While respiratory symptoms like cough, dyspnea, and fever are prevalent among individuals infected with SARS-CoV-2, also referred to as COVID-19, a clinically significant subset of patients with COVID-19 have also reported gastrointestinal (GI) symptoms. Diarrhea is the most common GI symptom observed in children and adults infected with SARS-CoV-2; this occurs because of viral spread from the infected to uninfected cells of the GI tract [3]. During SARS-CoV-2 infection, initially, a high viral load and reduced inflammatory activity are observed, in addition to certain symptoms associated with GI illness [4]. Although many studies have reported diarrhea as a symptom of COVID-19, a strict definition is missing [3,4,5]. The direct impact of SARS-CoV-2 on the gastrointestinal tract may contribute to diarrhea during infection, while its effects on the gastrointestinal tract can lead to bleeding and inflammation, further complicating the COVID-19 disease process in other organs [6,7].

Coronaviruses (CoVs) are RNA viruses belonging to the order Nidovirales in the subfamily Coronaviridae; they comprise enveloped and positive-stranded viruses [1]. Among the four types (α, β, γ, and δ) of coronaviruses, α- and β-CoVs have been identified as causative agents of both respiratory tract infections and gastroenteritis in both humans and animals [8]. Seven different human CoVs (HCoVs) have been identified: HCoV-NL63, HCoV-OC43, HCoV-229E, and HCoV-HKU1 are endemic viruses and SARS-CoV-1and Middle-East respiratory syndrome virus (MERS-CoV) is epidemic, and SARS-CoV-2 a pandemic virus. HCoVs are well recognized as the causative agent of respiratory illness. Besides the nasal mucosa, these viruses are capable of infecting multiple organs, including the colon, spleen, liver, lungs, and brain [2]. HCoVs may be present along the GI tract, particularly in the intestinal epithelial cells (at least in one intestinal segment), as evidenced by biopsies performed using endoscopy and infected patients presenting positive virus in their feces even after viral clearance from the respiratory tract [2,9,10].

The entry of SARS-CoV-2 infection involves binding to the angiotensin-converting enzyme 2 (ACE2) receptor on the surface of host cells [5,9]. Other than the lung, ACE2 is highly expressed in GI organs such as the esophagus, ileum, and colon, mainly in esophageal epithelial cells and absorptive enterocytes [11,12,13,14]. ACE2 expression is significantly more abundant in the GI system, particularly in the colon, by a factor of 100 in comparison to the respiratory system [14]. Specific organs exhibit increased susceptibility to infection due to their high abundance of ACE2 receptors. Notably, the epithelial cells lining the terminal ileum and colon display a particularly high expression of ACE2 receptors. This observation holds significance as mucosal inflammation, a characteristic feature of inflammatory bowel disease (IBD), is commonly observed in these specific intestinal regions among patients diagnosed with Crohn’s disease (CD) and ulcerative colitis (UC) [15,16]. Given the high number of ACE2-expressing organs in the GI system, it is not unexpected that this system is vulnerable to viral infiltration.

Emerging research indicates that COVID-19 can affect the gut–lung axis and lead to dysbiosis, a microbial imbalance in the gut. Moreover, higher mortality rates have been observed among elderly patients [17]. Changes in gut permeability and decreased epithelial integrity can also impact the severity of liver disease and metabolic diseases, inducing systemic inflammation. Pulmonary diseases have also been linked to disrupted gut microbiota and intestinal barrier [18,19]. In fact, COVID-19 patients with digestive symptoms are more likely to experience liver injury, underscoring the importance of maintaining a healthy gut microbiota. Therefore, understanding the interplay between SARS-CoV-2 and the gut microbiota is crucial for improving our understanding of the pathophysiology of COVID-19.

Increasing evidence suggests that SARS-CoV-2 infections affect multiple GI areas, possibly resulting in several symptoms associated with a dysfunctional GI system. In order to advance our comprehension of the correlation between SARS-CoV-2 and the GI tract, it is imperative to acquire a comprehensive understanding of the digestive manifestations and pathophysiology associated with these viruses. Therefore, this review summarizes the manifestations and potential mechanisms of GI injuries in patients with COVID-19.

## 2. Routes of Transmission of SARS-CoV-2

The transmission of all HCoVs most commonly occurs via respiratory droplets produced by infected individuals, direct contact with infected people and animals, and exposure to infected particles or surfaces [20,21,22,23,24]. However, epidemiological and virological studies and bioinformatics modeling suggest that HCoVs can be transmitted to humans via the GI tract and that viral RNA can be detected in the feces of many people with confirmed HCoV infection. Notably, HCoV-HKU1, HCoV-229E, HCoV-NL63, and HCoV-OC43 can be detected in the fecal samples of children with acute gastroenteritis [25,26]. Likewise, the infection has been documented in cases where actively replicating virus, and viral particles were detected in fecal samples of patients afflicted with MERS-CoV and SARS-CoV-1 [27,28]. These viruses are detected in the fecal samples within 14–21 days of disease onset, confirming the fecal-to-oral route transmission [23,29,30]. Although it has been demonstrated that SARS-CoV-2 RNA can be detected in feces, it is still unclear whether or not the virus can be transmitted through feces. Aerosols containing viral RNA have been found in surrounding toilet bowls, further confirming fecal-to-oral transmission of SARS-CoV-2 [31,32]. Even after the virus is cleared from respiratory samples, SARS-CoV-2 can still be found in feces [9]. Approximately 70% of the infected patients shed their viral RNA through the GI tract [33]. Significantly, the identification of subgenomic mRNAs (sg mRNAs) of SARS-CoV-2 in fecal samples from a patient provides evidence of actively infected cells within the GI system. Since sg mRNAs are not encapsulated within virions, they have the potential to withstand RNAse digestion and traverse the GI tract. Consequently, current research has demonstrated the recovery of viable SARS-CoV-2 from fecal specimens obtained from individuals experiencing severe cases of COVID-19 [31,34]; other studies have reported being unable to isolate infectious virions from fecal samples. Therefore, whether the presence of SARS-CoV-2 infectious virions in feces is a rare or frequent occurrence remains unknown. While HCoV-NL63, HCoV-OC43, and HCoV-229E have not been known to spread through the fecal–oral route, the presence of viral particles and the fecal–oral transmission has been established for viruses such as SARS-CoV-2, SARS-CoV-1, MERS-CoV, and HCoV-HKU1. Nevertheless, more studies are warranted to analyze the level of fecal-to-oral transmission.

## 3. Digestive Manifestations and Pathophysiology of SARS-CoV-2 Infection

SARS-CoV-2 is generally known to cause acute respiratory distress syndrome (ARDS); however, recent studies suggest that these viruses also cause gastroenteritis by replicating in human enterocytes. The GI symptoms associated with SARS-CoV-2 were anorexia (15.8–50.2%), nausea and vomiting (2.0–22.7%), abdominal pain (0.1–4.4%), and diarrhea (2.0–49.5%) [5,32,35]. The main symptoms observed in SARS-CoV-2-induced (GI) involvement are diarrhea, vomiting, nausea, and abdominal pain, and GI lesions are similar to the symptoms observed in SARS-CoV-2-induced GI manifestations [31]. The detection of SARS-CoV-2 RNA in feces suggests that the virus can multiply within the epithelial cells of the gastrointestinal tract [34,36]. Autopsy and biopsy studies on COVID-19 patients have reported several gastrointestinal changes, including necrosis, exfoliation of the epithelium of the esophagus, stomach, and intestine, stem cell degeneration, hepatic sinus congestion, hepatomegaly, and the infiltration of mononuclear cells and lymphocytes into the portal area. Leading to focal necrosis with neutrophilic infiltration [37,38]. A study reported that in patients with SARS-CoV-2 infection, diarrhea occurs before the manifestation of respiratory symptoms in 2.9–6.3% of patients [38]. GI symptoms expose patients with COVID-19 to electrolyte disturbances, including decreased sodium levels; this may, in turn, aggravate the disease [3]. In patients with gastroenteritis, the symptoms include severe abdominal pain (0.98–5.8%), nausea (1.0–27.5%), anorexia (10.1–39.7%), vomiting (1.0–12.5%), and diarrhea (2.0–55.0%) [31,39,40]. According to a recent study, approximately 79% of patients infected with COVID-19 exhibited GI symptoms at the onset of infection and during subsequent hospitalization [41]. On the other hand, in a case series at a UK center, GI symptoms such as abdominal pain, diarrhea, and dyspepsia were observed after COVID-19 infection [42]. Studies on the long-term effects of SARS-CoV-2 infection have revealed that the recovery from COVID-19 and its aftereffects is associated with long-term sequelae of physiological and GI dysfunction [43]. Individuals who have recovered from COVID-19 but continue to experience symptoms even after >12 weeks of the infection are said to suffer from “long COVID” [44,45]. Patients with long COVID continue to suffer from digestive symptoms such as abdominal pain and vomiting or develop digestive sequelae such as abdominal pain, acid reflux, abdominal distension, vomiting, belching, and rectal bleeding [46]. However, the presence of pre-existing chronic conditions has not been definitively established as a confirmed risk factor for persistent GI symptoms in patients with COVID-19. Nevertheless, it is important to note that these chronic conditions have been identified as risk factors for hospitalization and mortality.

Effenberger et al. [47] found that SARS-CoV-2 infection causes an inflammatory reaction in the GI tract, characterized by elevated levels of fecal calprotectin, which is a biomarker for intestinal inflammation primarily produced in neutrophil granulocytes, as well as a systemic IL-6 response. According to studies conducted by Hung et al. [6] and Tian et al. [41], severe COVID-19 patients who manifested gastrointestinal symptoms were also observed to have esophageal bleeding accompanied by erosions and ulcers, as well as notable changes in their intestinal histology. In addition, a considerable number of infiltrating plasma cells and lymphocytes accompanied by interstitial edema were observed in the lamina propria of the gastrointestinal tract among 73 COVID-19 patients who were hospitalized [6,41].

IBD and COVID-19 are two pathological conditions that seriously compromise the structure and function of the GI tract; they share, at least in part, several pathological mechanisms, including gut inflammation, mucosal hyperpermeability, intestinal barrier breakdown, gut bacterial overgrowth, dysbiosis, and changes in the blood and fecal metabolomes [48]. The two medical conditions classified under the umbrella of inflammatory bowel disease (IBD) are Crohn’s disease (CD) and ulcerative colitis (UC) [49]. Several investigations have explored the vulnerability of individuals with IBD to SARS-CoV-2 infection, the appropriate management of these patients during the COVID-19 outbreak, and the advantages and disadvantages of administering immunomodulatory therapy, specifically in children [50,51,52]. However, there is contradictory evidence regarding the effects of IBD on the incidence and outcome of COVID-19 [53]. Imperatore et al. [54] reported a case study involving a male patient who experienced severe abdominal pain and chronic bloody diarrhea following recovery from a SARS-CoV-2 infection. The patient was admitted to the hospital and underwent bowel sonography, endoscopy, and histological examination. The findings revealed a diagnosis of de novo colitis, as the patient had not exhibited GI symptoms during the SARS-CoV-2 infection and had not been previously diagnosed with inflammatory bowel disease (IBD). Recently, several studies have reported that SARS-CoV-2 infection may lead to a de novo UC in patients without GI symptoms before the infection [54,55]. The connection between COVID-19 and IBD was investigated in a recent meta-analysis, which analyzed the overall incidence of COVID-19, hospitalization due to COVID-19, severe COVID-19 cases, and mortality in patients with IBD [56], and identified notable correlations between UC and heightened susceptibility to COVID-19, COVID-19-related hospitalization, and severe cases in COVID-19 patients. Conversely, they also discovered that CD is noticeably linked with a reduced risk of severe COVID-19. One possible explanation for the increased hospitalization rate and severity of COVID-19 in UC patients compared to CD patients is the more severe form of the disease, greater degree of immunosuppression, and greater likelihood of hospitalization resulting from COVID-19. While COVID-19 typically presents with milder symptoms in children and infants, there is a possibility of a severe systemic inflammatory response known as multisystem inflammatory syndrome in children (MIS-C). MIS-C is characterized by persistent fever, inflammation, and severe illness that often requires hospitalization. Anomalies in the digestive system may be the cause of MIS-C. Interestingly, >80% of patients with MIS-C have GI symptoms [57]; on the other hand, only 10–15% of adults with COVID-19 have GI symptoms [50]. The occurrence of GI symptoms and the prolonged interval between infection and clinical manifestation in patients with MIS-C suggest that the underlying mechanisms contributing to GI pathology in these patients may differ from those observed in adults with active COVID-19. Notably, MIS-C associated with COVID-19 is frequently accompanied by GI symptoms such as diarrhea, nausea, and vomiting. These clinical observations suggest that the development of MIS-C involves biological mechanisms that are associated with intestinal mucosal dysfunction and disruption of the epithelial barrier. Yonker et al. [58] observed that children with MIS-C had increased levels of plasma zonulin, a protein that dilates the tight junctions in the intestinal and gastric walls, as well as detectable SARS-CoV-2, indicating ongoing viral replication in the intestines. Therefore, they proposed that intestinal barrier breakdown could lead to SARS-CoV-2 antigenemia, ultimately resulting in the development of MIS-C. Supporting this hypothesis, Yonker et al. [58] observed higher levels of the SARS-CoV-2 spike protein, particularly the SARS-CoV-2 spike 1 (S1) component, in the plasma of children with MIS-C than in those without MIS-C or healthy controls. Furthermore, they conducted a fascinating proof-of-concept experiment in which they administered larazotide, a zonulin antagonist, to a child with MIS-C which led to a marked improvement in the child’s clinical condition suggesting that therapeutic strategies aimed at reestablishing epithelial barrier integrity in SARS-CoV-2-infected children warrants further investigation [58]. 

## 4. Pathogenesis of SARS-CoV-2 in the GI Tract

SARS-CoV-2 primarily infects humans and leads to respiratory and GI tract discomfort, as well as systemic manifestations affecting multiple organs. However, in the case of several organs, it remains unclear whether the virus directly infects the organ or if the symptoms arise as a result of the lung’s side effects due to SARS-CoV-2 infection. Nevertheless, in the case of GI tract symptoms, the presence of the viral genome in the feces suggests that SARS-CoV-2 replicates in the epithelial cells [34,36]. In patients with SARS-CoV-2 infection, ACE2 and other receptors are downregulated in both infected and neighboring cells as well as in ileum-derived and colon organoids [2]. Clinical studies on several patients have revealed that viral entry does not involve the digestive system but that the virus enters through the epithelial cells of the intestinal mucosa [37].

Abdominal pain is a common symptom observed in severely infected patients [38], possibly resulting from the substantial infiltration of lymphocytes in the squamous epithelium of the rectum, stomach, and duodenum [3,33,59]. Further, SARS-CoV-2 infection alters intestinal membrane permeability, leading to enterocyte dysfunction [4]. In addition to enterocyte dysfunction, intestinal inflammation and diarrhea also suggest the pathological importance of ACE2 expression in the epithelial cells of the small intestinal, which are more vulnerable to SARS-CoV-2 infection [60]. In patients with SARS-CoV-2 infection, stress-induced gastric mucosal damage occurs owing to multiorgan failure, hypoxia, disease severity, mechanical ventilation, ARDS, and psychological stress [61]. Patients with SARS-CoV-2 infection frequently display increased levels of calprotectin, a biomarker released by neutrophils during inflammation of the GI tract, along with increased levels of serum IL-6 [4]. The infection-induced cytokine storm triggers secondary hemophagocytic lymph histiocytosis, a hyperinflammatory disease linked to multiorgan failure and high mortality rates in COVID-19 patients [3,62,63]. In addition, there are many clinical similarities between MIS-C and toxic shock syndrome (TSS), which is induced by bacterial superantigens (SAgs) [64]. Owing to the striking similarities between MIS-C and TSS, SARS-CoV-2 was studied to identify the presence of SAg-like structures; it was found that the S1 glycoprotein contains a novel SAg-like motif that is highly similar to a Staphylococcal enterotoxin B fragment. Bioinformatics analyses revealed a strong level of specificity in the binding of the SAg-like motif to T-cell receptors (TCRs) and major histocompatibility complex class II proteins. The notable increase in the expression of a TCR β variable gene in peripheral blood samples obtained from MIS-C patients aligns with a SAg-induced immune response. It is associated with the severity of MIS-C and serum cytokine levels. Finally, children with MIS-C have increased levels of circulating S1, increased gut permeability, and prolonged persistence of SARS-CoV-2 RNA in the gut [64]. 

Having to deal with trillions of microorganisms, the intestinal epithelium has developed a mechanism to generate antimicrobial peptides, such as HD5, a peptide with lectin-like properties that can bind to lipid-glycosylated proteins [64]. The production of HD5 is abundant in the small intestine’s Paneth cells and in the neutrophils’ granules [64,65]. It inhibits viral entry into the enterocyte by binding to the ACE2 receptor, thereby blocking several sites of the ligand binding domain of the SARS-CoV-2 spike protein. HD5 exhibits a higher binding affinity to ACE2 than HD5 and the other intestinal α-defensin HD6 [64]. Similarly, the entry of SARS-CoV-2 into Caco-2 cells was inhibited using spray-dried HD5 powder [65]. The secretion of antimicrobial peptides from Paneth cells, which harbor a compositional diversity of bacteria, as well as the release of cytokines (IL-2, IL-7, TNF-α, interferon-gamma (IFN-γ) inducible protein, and macrophage inflammatory protein1-α) results in convalescent stages in patients with diarrhea [14,66,67]. Recently, Yantiss et al. [58] have employed various techniques such as immunohistochemical analysis, in situ hybridization for spike protein, and ultrastructural visualization of viruses within the intestinal epithelium to confirm the presence of SARS-CoV-2 infection. They observed cytoplasmic blebs and tufted epithelial cells in the infected area, but no inflammation was detected. Additionally, the study found that even after the virus was undetectable, it could still result in gastrointestinal symptoms. The authors suggest that the damage to the epithelial cells and the subsequent diarrheal symptoms may be due to systemic cytokine and complement cascades rather than direct viral injury [68]. These observations align with existing clinical and in vitro data and expand our knowledge of the various manifestations of COVID-19. These findings imply that SARS-CoV-2 may cause severe harm to the human GI tract distinctly from the respiratory system, potentially disrupting the microbial balance and contributing to the development of different diseases.

## 5. Role of Receptors in SARS-CoV-2 Pathogenesis

SARS-CoV-2 attaches to its host cell via interactions between the spike glycoprotein (S) and ACE2 [2,8,9,10]. Before the release of SARS-CoV-2 S protein into the cytosol of host cells, it undergoes processing by a plasma membrane-associated type II transmembrane serine protease (TMPRSS2) following its binding to the ACE2 receptor. Glandular epithelial cells in the GI tract, specifically those in the gastric, duodenal, and rectal regions, display elevated levels of ACE2 receptor expression. However, the ACE2 receptor is not expressed in the colon [34,69]. Comparative and differential expression pattern analyses of ACE2 across >150 different cell types representing all major human tissues and organs revealed that ACE2 expression was highest in enterocytes, including those in the small intestine, colon, and duodenum [70]. Single-cell sequencing revealed higher expression of ACE2 in intestinal epithelial cells than in lung cells [2,9,71]. The messenger RNA for ACE2 is stabilized by the neutral amino acid transporter B0AT1, a member of solute carrier family 6 (SLC6A19) [72], present in the GI system; therefore, it is a prerequisite for SARS-CoV-2 infection. ACE2 is required as a functional partner for the expression of B0AT1, on the luminal surface of intestinal epithelial cells [59]. Furthermore, it has been reported that transmembrane serine protease 2 (TMPRSS2) plays a crucial role in facilitating the entry of enteric viruses, including SARSCoV-2, into host cells [73]. Furthermore, Ning et al. [26] utilized shotgun proteomics with tandem mass tags to show that ACE2 levels were higher in inflamed regions of the intestines among CD patients as opposed to UC patients.

A study reported that SARS-CoV-2 exhibits nearly 20 times greater affinity to ACE2 than other SARS-CoV-1s that bind to the ACE2 receptor [23]; this demonstrates the extremely contagious nature of SARS-CoV-2. In the small intestine, ACE2 is particularly present in proximal and distal enterocytes [10]. The jejunum has the highest expression of ACE2 and TMPRSS2. Further, ACE2 is abundantly expressed in ciliated cells. The expression of the ACE2 receptor during viral infection greatly varies among different individuals [74]. The epithelial cells in the ileum express high levels of ACE2 in the absorptive enterocytes and low levels of ACE2 in the progenitor absorptive cells [35]. In contrast, the expression of the increased TMPRSS2 levels observed in the colon and ileum could potentially aid the viral invasion of enterocytes in the GI [10]. Analysis of single-cell transcriptomes unveiled the co-expression of ACE2 and TMPRSS2 in various cell types, including upper esophageal cells, upper epithelial cells, gland cells, and absorptive enterocytes in the ileum and colon [61]. Further, ACE2 and TMPRSS2 are co-expressed in huge amounts in small intestinal enterocytes (20%) and colon cells (5%) [75]. They also found that immature enterocytes 2 are the primary targets for SARS-CoV-2 in the human intestinal epithelial cells in both the colon and ileum. However, these cells do not exhibit the highest expression levels of ACE2. However, the expression level of TMPRSS2 is high in immature enterocytes 2. This implies that the degree of susceptibility to viral infection is not strongly linked to the expression level of ACE2 [2]. Nonetheless, the co-expression of ACE2 and TMPRSS2 in the enterocytes is crucial for the entry of SARS-CoV-2 into host cells [73,75]. While SARS-CoV-2 employs various receptors for binding, the ACE2 receptor is prominently expressed in the GI tract. This abundance of ACE2 receptors in the GI tract suggests that the virus has the potential to induce digestive manifestations.

**Figure 1:**
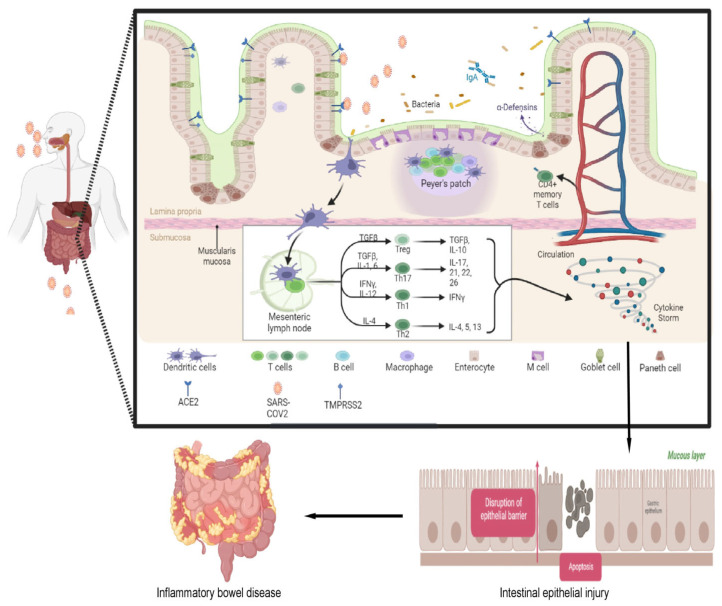
The impact of SARS-CoV-2‘s affinity for the intestinal tract on mucosal immune response and its potential role in the initiation or exacerbation of IBD. The interaction between the spike protein of SARS-CoV-2 and ACE2 receptors on enterocytes facilitates viral entry into the gut, while TMPRSS2-mediated spike protein processing contributes to viral internalization. In response to SARS-CoV-2 infection, the body’s innate and adaptive immune systems become overly active, leading to an increase in the number of activated immune cells. Excess cytokines are produced through a cascade reaction, resulting in a cytokine storm, and IL17 secreted by Th17 cells plays a crucial role in this. The release of diverse proinflammatory mediators damages the intestinal epithelial cells, culminating in the aggravation or onset of IBD.

SARS-CoV entry relies, in addition to ACE2, on TMPRSS2/TMPRSS4 because the latter assists in the cleavage of the S protein of the virus on the cell membrane, thereby facilitating viral fusion with the cell membrane [74]. In the absence of TMPRSS2, cathepsin L is responsible for cleaving the S1/S2 polybasic cleavage site to activate the SARS-CoV-2 spike protein in endosomes [73,76,77]. To evade detection by the host system, SARS-CoV-2 can potentially enter the host using either a clathrin-dependent or clathrin-independent endocytosis pathway [33,78]. Recently, a study demonstrated that besides fusion with the plasma membrane, SARS-CoV-2 also enters the host cell through the novel clathrin- and caveolae-mediated endocytic pathway [79]. The S protein regulates the fusion of SARS-CoV-2 with the host cell membrane, an essential step in cellular uptake. Host trypsin-like proteases regulate the activation of the S protein via proteolytic cleavage; their activity is upregulated in patients with IBD. The activation of the S protein is also regulated by host trypsin-like proteases through proteolytic cleavage, and their function is increased in IBD patients [16,80]. This upregulation may promote viral infection in IBD patients. Notably, ACE2 protein expression was found to be higher in the colon and terminal ileum of patients with IBD compared to noninflamed subjects [16]. Furthermore, when compared to both healthy individuals and patients with active inflammatory bowel disease (IBD), patients with IBD exhibited higher ACE2 activity in the noninflamed colon. Moreover, the plasma of patients with IBD displayed a higher ACE2: ACE ratio compared to individuals without IBD. Notably, patients with IBD, particularly those with Crohn’s disease (CD), demonstrated elevated levels of soluble ACE2 expression [16]. Due to ACE2‘s ability to modulate the inflammatory immune response, COVID-19 may impact immune-inflammatory diseases such as IBD that affect the ileum by interfering with ACE2 [81]. Taken together, these observations suggest that individuals with IBD may be more vulnerable to COVID-19. However, at present, there is no evidence to support this notion. The pictorial representation of SARS-CoV-2 causing or exacerbating IBD by modulating mucosal immune response is depicted in Figure 1.

## 6. Immune Response of the Host to SARS-CoV-2 Infection

The progression of the disease and elimination of the virus is influenced by the innate and adaptive immune response of the host to the viral infection. The initial line of protection against viruses is the innate immune system [82]. The release of proinflammatory cytokines and chemokines into the bloodstream due to the stimulation of innate and adaptive immune cells can impact the gut microbiota [4]; this alteration of the gut microbiome is called gut dysbiosis [83,84]. During secondary infection by CoVs, the initial phase of respiratory symptoms escalates, while intestinal symptoms subside; nevertheless, cytokine-induced inflammation worsens. In response to SARS-CoV-2 infection, the innate and adaptive immune systems of the infected host are activated. This leads to the release of mediators and chemokines by infected cells, which in turn causes the localized accumulation of neutrophils at the site of infection. Infection with SARS-CoV-2 stimulates the activation of both the innate and adaptive immune systems in the host. As a result, the infected cells release mediators and chemokines, leading to the accumulation of neutrophils at the site of infection. The accumulated neutrophils perform antiviral effector functions and secrete chemokines and cytokines to attract other immune cells, including T lymphocytes and monocytes [3]. Infected patients with gastroenteritis exhibit higher neutrophil counts, an elevated neutrophil–lymphocyte ratio, lower lymphocyte counts, elevated C-reactive protein levels and decreased eosinophil, monocyte, and basophil counts [3,4] Neutrophil recruitment also occurs when a disturbance in the intestinal flora promotes Th17 cell polarization in the small intestine and the production of excessive IL-17A [85].

## 7. Innate Immune Responses in the Gut during SARS-CoV-2 Infection

The mucosa’s initial antiviral innate response is followed by a potent and efficient adaptive immune response. Mucosa-associated lymphoid tissue (MALT) in the large intestine and gut-associated lymphoid tissue (GALT) in the Peyer’s patches of the small intestine harbor microfold cells (M cells). M cells, by transporting antigens from the intestinal lumen to immune cells, contribute to the initiation of region-specific immune responses. Following this, immune cells, along with antigen-presenting cells, migrate to a specialized pocket situated on the surface of the basement membrane of M cells. During excessive viral replication, M cells get destroyed, leading to inflammation and diarrhea, and thus SARS-CoV-2 manages to evade immune detection by not presenting antigens to lymphoid follicles [86]. As a result, direct infection of enterocytes can lead to cytokine release and the immune response weakens. This allows viral particles in the lumen to infect more enterocytes, resulting in further damage to the GI tract. The inflammation caused by cytokine release is regulated by the intestinal signaling pathway via dendritic cells [4]. As a result, inflammatory cells are recruited; this, in turn, results in the production of inflammatory cytokines in intestinal epithelial cells [87].

The innate immune system detects viral materials that may be pathogenic and signals to the nucleus to express types I and III interferons (IFNs) and other proinflammatory cytokines. IFNs suppress viral replication and reduce the number of infected cells by activating a cascade of cellular and molecular events. In SARS-CoV-2 infection, 36 genes were reported to be involved in the immune pathways and cytokine signaling, i.e., the IFN-γ signaling pathway [4]. Type I IFNs are secreted in the innate immune response, whereas type III IFNs are secreted in the intrinsic immune response, with the latter capable of acting on intestinal epithelial cells, reinforcing an antiviral state against the infection [2]. The release of cytokines from the human epithelial cells of the lamina propria may serve as an alternate source of the cytokine storm in the lungs [2]. Upon SARS-CoV-2 infection, the intestinal epithelium and colon organoids exhibit a distinct antiviral response characterized by the secretion of type III interferons (IFNs), in contrast to the airway epithelium. Moreover, human intestinal organoids are capable of producing both type I and type III IFNs upon enteric viral infection [2]. IFNs, the most common antiviral component, are also produced by the human intestinal epithelial cells upon SARS-CoV-2 infection. They induce the IFN-stimulated genes (ISGs) in a paracrine manner to control viral infection in neighboring cells. SARS-CoV-2 is capable of blocking IFN signaling [2]. Further, Triana et al. [88] reported that human intestinal epithelial cells generate strong proinflammatory and IFN-mediated responses upon SARS-CoV-2 infection, as revealed by comparative gene expression analyses. Several gene expression and pathway analyses in this study confirmed that the colon-derived and ileum-derived immature enterocytes 2 exhibit a robust NF-κB/TNF-mediated response. Conversely, neighboring immature enterocytes 2 demonstrate an IFN-mediated response, resulting in the expression of ISGs in a paracrine manner. Moreover, the study found that organoids derived from the ileum exhibit a stronger immune response compared to those derived from the colon. 

**Figure 2:**
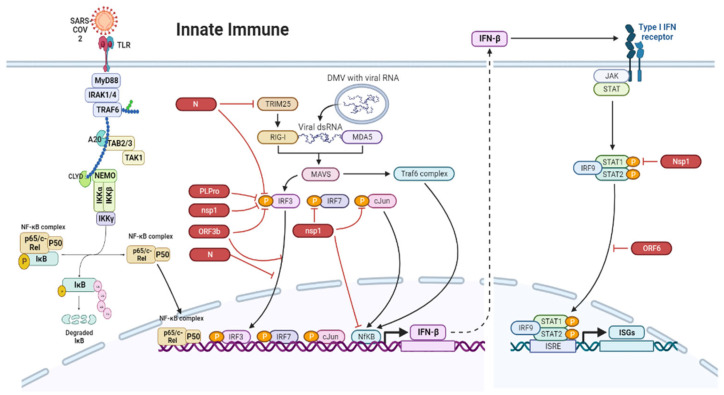
SARS-CoV-2 employs different mechanisms to activate the host’s innate immune system. Toll-like receptors (TLR) recognize viral RNA and activate the NFκB transcription factor to induce the expression of inflammatory cytokines and type I interferons. TLR’s cytoplasmic domain recruits the adaptor protein dMyD88, which triggers a signaling pathway that eventually releases NFκB from its cytoplasmic inhibitor. Once released, NFκB translocates into the nucleus where it activates transcription of genes encoding proinflammatory cytokines and induces IFN gene expression. The interferon regulatory factor (IRF) proteins remain inactive in the cytoplasm until they are phosphorylated. Once activated, they translocate to the nucleus as active transcription factors. IRF3 and IRF7, two of the nine members in the IRF family, are vital for the expression of antiviral type I interferons and play a critical role in TLR signaling. The Janus kinase and signal transducer and activator of transcription (JAK-STAT) pathway is a rapid signaling pathway utilized by numerous cytokine receptors to activate the transcription of interferon-stimulated genes (ISGs). Coronaviruses use papain-like protease (PLpro), NSP1, ORF3b, and N protein to evade the host innate immune system by inhibiting two transcription factors, IRF3 and IRF7, which are necessary for IFN transcription.

In SARS-CoV-2 infection, viral replication is reduced by the recognition of the virus by the innate immune system, which produces type II IFNs and the cytokines IL-4, IL-10, and IL-6 [37,88]. In the early stage of SARS-CoV-2 infection, there is a delayed release of chemokines and cytokines from dendritic cells and macrophages; this is followed by the release of small amounts of antiviral IFNs and large amounts of proinflammatory cytokines and chemokines [32]. Because of the gut–lung axis, the gastric and respiratory flora could influence each other, and any disorder in the flora could be bidirectional [37,89,90]. Further, the gut–lung axis may be influenced by intestinal mucosal damage and bacterial imbalance [37]. To evade recognition by the host’s innate immune system, several viral proteins of coronaviruses, such as papain-like protease (PLpro), NSP1, ORF3b, and N, inhibit two crucial transcription factors (IRF3 and IRF7) that are required for the transcription of IFN [67]. the structural proteins of SARS-CoV, such as NSP1, ORF6, and N proteins [91,92] block the IFN and NF-kB pathways by binding to the signaling proteins in these pathways. ORF6 mainly blocks the nuclear import protein and enhances SARS-CoV replication [91]. Moreover, ACE2 regulates innate immune responses and influences the composition of the intestinal microbiota of the host [93]. Different mechanisms by which SARS-CoV-2 activates the innate immune system in the intestine are depicted in Figure 2.

A study reported that active replication of SARS-CoV-2 is required for immune responses [88]. The signaling pathway data from single-cell RNA sequencing were analyzed to identify the signaling pathways in the infected and neighboring cells. Upon closer investigation of the signaling pathways, it was revealed that neighboring cells displayed the activation of transcription factors IRF1 and STAT1, whereas infected cells exhibited activation of cJun. Importantly, despite the presence of IFN, cells infected with SARS-CoV-2 did not show upregulation of the JAK-STAT pathway or expression of ISG [88]. These signaling pathways were confirmed by the expression of ISG and NF-κB and inhibition of HSP90 using the Calu-3 and Caco-2 cell lines. As a result, the inflammation level in the patients was directly proportional to the degree of the GI lesions. In case of a severe SARS-CoV-2 infection, the patients secreted higher levels of IL-10, IL-7, IL-2, and granulocyte colony-stimulating factor in the plasma and had elevated levels of monocyte chemoattractant protein-1 (MCP-1), INF-γ-induced protein-10, TNF-α, and macrophage inflammatory protein-1A. Patients with a severe SARS-CoV-2 infection exhibit increased secretion of IL-10, IL-7, IL-2, and G-CSF in their plasma, alongside increased levels of monocyte chemoattractant protein-1 (MCP-1), IFNF-γ-induced protein-10, TNF-α, and macrophage inflammatory protein-1A [37,94]. In addition, elevated levels of hyperinflammation markers, including procalcitonin, C reactive protein, D-dimer, and ferritin, are observed in patients with SARS-CoV-2 infections [3,95]. The inflammatory response of the gut mucosa can alter the intercellular space of enterocytes, leading to an increase in intestinal permeability. Consequently, bacterial antigens and toxins may translocate into the bloodstream, exacerbating the disease state of COVID-19 patients. 

The Notch pathway plays an important role in regulating innate immunity and inflammation [96,97]. Notch pathway elements can be affected by viral proteins, leading to abnormal functions [98], and the upregulation of NOTCH signaling signatures was substantiated in the lungs of SARS-CoV-2-infected rhesus macaques [99]. Therefore, targeting Notch may prevent SARS-CoV-2 infection [99]. Upon interaction with its ligand, the transmembrane receptor undergoes successive proteolytic cleavages mediated by the ADAM10 or ADAM17 protease and the γ-secretase complex, resulting in the transcription of Notch target genes, including furin and miRNA-145 [99]. Furin has been shown to be essential for viral entry into cells, in addition to ACE2 [100]. Notch1 induces the transcription of furin, and inhibiting Notch1 may decrease furin levels, potentially interfering with the virus’s ability to enter cells [99,101]. The proteolytic cleavage of ACE2 and competition with TMPRSS2 both need ADAM17, which is highly expressed in the heart and lungs [101,102,103]. The Notch pathway positively regulates furin and negatively regulates ADAM17 [103]. However, there is conflicting information on the role of ADAM17-mediated cleavage in SARS-CoV-2 entry [102,104].

Elevated levels of serum interleukin 6 (IL-6) were observed in COVID-19 patients. The immune response in these patients is triggered and sustained by the interplay between Notch signaling and IL-6, which leads to a cytokine storm [99]. Delta-like ligand 4 (Dll4)-induced Notch signaling in macrophages causes an increase in IL-6 production, and IL-6 upregulates the expression of Notch ligands like Dll1 and Dll4 [105]. This feedback loop further drives the expression of IL-6. Disrupting the positive feedback loop responsible for the cytokine storm seen in COVID-19 could be achieved by targeting the Notch pathway and inhibiting Dll ligands. Furthermore, there is a relationship between Notch and immune response, with SARS-CoV-2 causing a cytokine storm in COVID-19 patients. Therefore, inhibiting Notch could be a therapeutic strategy for preventing SARS-CoV-2 infection and relieving COVID-19 symptoms [106].

## 8. Adaptive Immune Response

While there is a growing body of research on the effect of SARS-CoV-2 infections on pulmonary and systemic immune responses [107,108], there is a dearth of data on intestinal immune responses. Immunohistochemistry and mass cytometry analyses conducted on intestinal tissue obtained from COVID-19 patients have revealed a notable elevation in intraepithelial CD8^+^ lymphocytes and CD4^+^/CD8^+^ effector T cells in the lamina propria when compared to healthy individuals [109]. On the other hand, a significant decrease in inflammatory dendritic cells within the lamina propria was observed in COVID-19 patients who exhibited GI symptoms [9]. In addition, Livanos, et al. [9] uncovered a substantial downregulation in pathways related to antigen processing, TH17 cell differentiation and reduced levels of key inflammatory proteins, including IL-6, CXCL8, IL-17A and CCL28 in circulation. These results suggest that SARS-CoV-2 infection in the intestines may modify the composition of systemic immune cells by transforming proinflammatory immune signatures into a more favorable immune environment. This phenomenon may account for the notable decrease in levels of IL-6 and IL-17 in COVID-19 patients experiencing GI symptoms, resulting in reduced mortality rates compared to COVID-19 patients lacking these symptoms.

It is possible that persistent SARS-CoV-2 infection in the intestines plays a crucial role in generating and sustaining a stable anti-SARS-CoV-2 antibody response by B cells. In spite of the absence of SARS-CoV-2 mRNA in nasopharyngeal swabs, Gaebler et al. [110] observed that SARS-CoV-2 virus particles and mRNA were potentially detectable in duodenal and ileal biopsies of COVID-19 patients even after a period of 3 months from the initial onset of the disease. Based on the data, it appears that SARS-CoV-2 viral reservoirs in the intestines can stimulate the production of long-lasting neutralizing anti-viral IgA antibodies. However, the levels of IgM and IgG antibodies targeting the SARS-CoV-2 spike protein receptor binding decrease six months after infection [110]. The prolonged existence of IgA antibodies may be particularly significant for maintaining long-term immune responses, as IgA dimers have demonstrated the ability to effectively neutralize SARS-CoV-2 [111]. These findings indicate that the presence of persistent viral infection in the intestines may play a crucial role in generating and sustaining effective immune responses against SARS-CoV-2.

A severe infection with SARS-CoV-2 can cause the death of activated immune cells. In patients with SARS-CoV-2 infection, reduced numbers of natural killer cells, B cells in the lower range, lymphocytopenia, a normal CD4^+^/CD8^+^ ratio, and decreased numbers of effector regulatory cells (CD25^+^CD127^+^) and memory T cells (CD45RO^+^) were observed [3]. Concurrently, there was an increase in immunological parameters such as IL-8, IL-10, IL-6, IFN-γ-inducible protein-10/CXCL-10, and MCP-1. Moreover, deviations in the frequencies of plasmacytoid dendritic cells (pDCs) and conventional dendritic cells (cDCs) have been noted [9]. As the next line of response, CD4^+^ T cells mediate protective and humoral immunity so that B cells produce virus-specific antibodies. Finally, to limit local infection, CD8^+^ T cells exhibit a cytotoxic function [3]. The presence of effector CD4^+^ T cells in the intestinal mucosa plays a critical role in both mucosal immunity and the development of chronic enteritis. Further, the C-C chemokine receptor type 9 (CCR9) is crucial for the entry of the CD4^+^ T cells into the small intestine and for interplay between CCR9 and CD4 in terms of SARS-CoV-2 [37]. CCL25 expressed in the epithelial cells of the small intestine can attract CCR9^+^ CD4^+^ T cells, which could compromise the intestinal immune system and modulate the homeostasis of the gut microbiome [37,112].

The levels of CD4^+^ T cells are reduced, and the distribution of naive and memory CD8^+^ and CD4^+^ cells influence the decrease in naive T cells [9]. The myeloid cells of the lamina propria exhibit reduced levels of cDCs, pDCs, and CD206^+^CD1c^+^ cells and increased levels of effector CD4^+^ T cells, CD8^+^ T cells, CD8^+^CD103^+^ T cells, CD4^+^CD103^+^ T cells, CD4^−^ CD8^−^ T cells, neutrophils, eosinophils, and IgM^+^ plasma cells [9]. 

**Figure 3:**
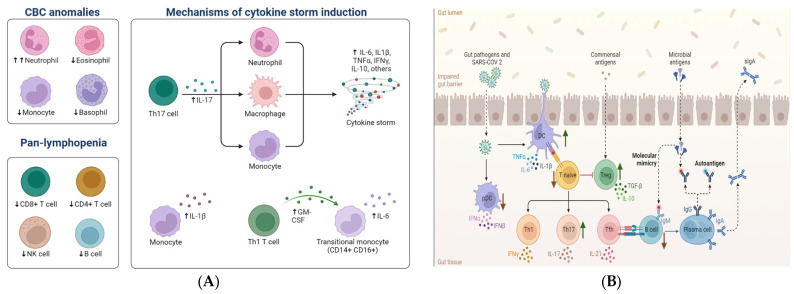
Immune cell responses in the innate and adaptive immune systems to SARS-CoV-2 infection. (**A**) SARS-CoV-2 infection can cause the death of activated immune cells. Reduced numbers of natural killer cells, B cells, lymphocytopenia, a normal CD4^+^/CD8^+^ ratio, and decreased numbers of effector regulatory cells and memory T cells have been observed in patients with SARS-CoV-2 infection. Simultaneously, levels of inflammatory cytokines IL-8, IL-10, IL-6, IFN-γ-inducible protein-10/CXCL-10, and MCP-1 were increased. (**B**) SARS-CoV-2 has significant impacts on the immune system by reducing plasmacytoid dendritic cells (pDCs) but increasing conventional dendritic cells c (DCs). SARS-CoV-2 causes the death of activated immune cells, leading to reduced B cells, natural killer cells, and lymphocytopenia. Effector regulatory and memory T cells also decrease, suppressing adaptive immunity. Inflammatory cytokines IL-8, IL-10, IL-6, IFN-γ-inducible protein-10/CXCL-10, and MCP-1 increase, worsening disease severity. Owing to SARS-CoV-2 infection, changes in the gut microbiome occur as a result of the release of proinflammatory cytokines and chemokines into the circulatory system, leading to gut dysbiosis. As a result of inflammation, the symbiotic microbiota that typically inhabits the gut is disrupted, leading to an overgrowth of a dysbiotic microbiota.

Key inflammatory genes, genes associated with inflammatory dendritic cells, and the host receptor neuropilin-1 (NRP1) are downregulated in the lamina propria of the intestine [9]. The upregulated genes involved in the immunomodulation of the lamina propria and epithelial cells include the genes for the antimicrobial peptide lipocalin-2, metallothionein, and transmembrane protein. Additionally, there is an elevation in the levels of oligoadenylate synthetase (OAS), a viral double-stranded RNA (dsRNA) sensor that possesses antiviral activity, in the lamina propria. The canonical antiviral genes and OAS3 in the epithelial cells are also upregulated. Conversely, the genes encoding heat shock proteins HSPA1A and HSPA1B are downregulated. Epithelial cells have decreased CD206^+^ T cells and increased CD4^−^CD8^+^ and CD8^+^ T cells. Certain inflammatory cytokines and chemokines (IFN-γ, IL-1b, CXCL8, and CCL24) are downregulated in the intestine of patients with SARS-CoV-2 infection, while CCL25 is upregulated, which is structurally similar to an antimicrobial peptide. Furthermore, there is a non-significant elevation in specific subsets, namely CD8^+^ T cells, intraepithelial lymphocytes, and CD8^+^CD69^+^ T cells, accompanied by the activation of (CD29^+^39^+^) CD4^+^ T cells in peripheral blood [9]. Symptomatic patients exhibit decreased levels of IL-17, IL-6, and CCL28 in their circulation. Taken together, the results suggest that the immune system activates ILs, IFNs, cytokines, chemokines, T and B cells, and white blood cells against SARS-CoV-2. An augmented effector function of Th17 cells, as well as other T cells in the gut mucosa, has been detected in COVID-19 infection. Consequently, SARS-CoV-2 has developed mechanisms to evade the innate antiviral response by modulating signaling pathways at various stages, thereby suppressing innate immune responses. More research will be necessary to determine if COVID-19 can also induce long-lasting T-cell memory responses or if it merely shares the intestinal persistence of SARS-CoV-2. Important aspects of SARS-CoV-2 infections, including localized immune reactions and gut dysbiosis in the intestine, are depicted in Figure 3A,B.

## 9. COVID-19 and Gut Microbiota

Normally, the GI tract harbors a highly diverse and complex microbial ecosystem that interacts with the host to maintain immune homeostasis [113,114,115]. Healthy gut microbiota contains *Faecalibacterium* spp., *Bifidobacterium* spp., *Ruminococcus* spp., and *Prevotella* spp., the latter being associated with low systemic inflammation [4,116]. A case study in Wuhan reported that the gut microbiome of healthy people is correlated with the susceptibility to contracting COVID-19 [117]. The study found that higher levels of Lactobacillus were associated with increased levels of the anti-inflammatory cytokine IL-10, better disease outcomes, and elevated levels of proinflammatory cytokines and bacterial species such as *Streptococcus*, *Klebsiella*, and *Ruminococcus gnavus* [117]. Patients with conditions such as IBD, obesity, and diabetes have exhibited an increased abundance of certain bacterial species in their gut microbiota [117].

In the presence of gut dysbiosis and inflammation, ACE2 levels may rise, impacting innate immunity, dietary amino acid equilibrium, and the microbial ecology of the gut [117]. In patients infected with the virus, the microbiota interacts with the viral particles, resulting in positive and negative consequences [118]. The surfaces of the bacterial envelope containing lipopolysaccharides in gram-negative bacteria and peptidoglycan in gram-positive bacteria have a positive correlation with viral proteins, probably to enhance receptor affinity of the latter and their entry into the host cells or for other similar mechanisms to facilitate infection in vivo [119]. Significant changes in the gut microbiota, called gut dysbiosis, occur because of the IFNs released by the viral molecules. These IFNs deplete the obligate anaerobic bacteria *Bifidobacterium*, *Lactobacillus*, and *Eubacterium* and promote the pathogenic bacteria *Corynebacterium* and *Ruthenibacterium* [117,120]. The fecal samples of patients with COVID-19 contained Bacteroides species capable of downregulating ACE2 expression in the murine gut; this alteration could increase the accessibility of the intestinal epithelial cells to SARS-CoV-2 [121], and the bacterium *Firmicutes* (*Erysipelotrichaceae*), which demonstrates a positive correlation in the murine gut. Gut dysbiosis was also detected in fatal SARS-CoV-2 cases with immune depletion and hypoxemia. Notably, gut dysbiosis is also observed in patients with obesity, diabetes, and autoimmune- and aging-related diseases [122]. ACE2 has been implicated in the modulation of the gut microbiota, as demonstrated by a study involving ACE2-KO animals. These animals exhibited altered gut microbiota, decreased expression of AMP, and reduced levels of amino acids like tryptophan. ACE2 facilitates the absorption of neutral amino acids such as tyrosine, valine, leucine, and tryptophan in the intestine [123,124]. The binding of SARS-CoV-2 to ACE2 receptors in the bowel reduces the number of free receptors available and disrupts the absorption of tryptophan, resulting in malabsorption and dysbiosis of the gut microbiota.

COVID-19-induced diarrhea may be caused by decreased ACE2 expression, reduced tryptophan absorption, gut dysbiosis, and dysregulated gut microbiota. The downregulation of ACE2 expression in infected enterocytes leads to intestinal dysbiosis. Dysbiosis, in turn, activates intestinal autophagy by inhibiting the mechanistic target of rapamycin [125,126]. As a result, NHE3, a protein that functions as a brush border Na^+^/H^+^ exchanger and controls NaCl absorption in the intestine [127], is degraded by autophagy. An increase in intestinal fluids and a secretory type of diarrhea are caused by decreased NHE3 levels [128]. Gut microbiota dysbiosis in patients with COVID-19 is mainly associated with changes in the abundance of *Bacteroides* spp. (*Bacteroides thetaiotaomicron*, *Bacteroides dorei*, *Bacteroides ovatusis*, and *Bacteroides massiliensis*) because these are associated with the attenuation of ACE2 expression in the gut. Therefore, changes in microbial composition may increase the accessibility of intestinal epithelial cells to SARS-CoV-2 [129].

In patients with SARS-CoV-2 infection, *Ruminococcus gnavus* exhibited the highest positive correlation with inflammatory markers, followed by an abundance of *Clostridium hathewayi Clostridium ramosum* and *Coprobacillus* Sp., which correlated with COVID-19 severity; further, an inverse correlation was observed with the abundance of anti-inflammatory bacterium *Faecalibacterium prausnitzii* [4]. The gut contains bacterial species capable of fermenting amino acids, including arginine [126]. These alterations in the gut microbiota composition can lead to an increased abundance of L-arginine-fermenting bacteria, resulting in a further decrease in the levels of free L-arginine. By increasing myeloid-derived suppressor cell numbers, impeding local T cell function, and increasing ROS generation, L-arginine depletion in the intestines can amplify intestinal inflammation [130].

The microbes residing in the gut can regulate mucosal sites located outside the intestine via the metabolites they produce, including short-chain fatty acids (SCFAs). These SCFAs can enter the bloodstream and influence other organs, playing a role in immune regulation and inducing the production of immunoglobulins while also exhibiting anti-inflammatory effects [35]. Fecal samples with high SARS-CoV-2 load specifically contain certain bacterial species, namely, *Morganella morganii*, *Streptococcus infantis*, *Collinsella aerofaciens* and *Collinsella tanakaei*, and [4]; in contrast, fecal samples with low to no SARS-CoV-2 load contain SCFA-producing bacteria, namely, *Alistipes onderdonkii*, *Bacteroides stercoris*, *Lachnospiraceae bacterium*, and *Parabacteroides merdae* [4]. An altered gut microbiota can promote the growth of SARS-CoV-2 and trigger the production of inflammatory cytokines, causing a “cytokine storm” that can damage tissues, trigger septic shock, and lead to multiorgan failure [37].

The administration of probiotics, such as lactic acid bacteria or bifidobacteria, has the potential to alleviate diarrhea symptoms and enhance the production of antiviral antibodies [37]. Further, the abovementioned probiotics may provide the required nutritional support to restore intestinal flora balance after microbial dysbiosis in the intestine owing to weak immunity [83,129]. The abundance of the opportunistic pathogens *Corynebacterium* and *Ruthenibacterium* was elevated, and a change in the fecal microbial profile was also observed [129]. Studies have reported a relationship between bacterial surface molecules and SARS-CoV infection. For example, the peptidoglycan of *Bacillus subtilis* has reduced SARS-CoV infectivity owing to the presence of surfactin, a cyclic lipopeptide responsible for CoV inhibition by disrupting the integrity of viral particles [131]. This study highlights the significance of the microbiota in viral pathogenesis and therapeutic interventions. The frequent utilization of antibiotics, such as penicillin, cephalosporins, macrolides, and quinolones, disrupts the gut microbiota and elevates the risk of lung cancer in humans [111,116]. In the COVID-19 diagnosis in China, the intestinal microecological balance is maintained using microecological regulators that help prevent secondary bacterial infections [37]. The emergence of new strains can have a considerable impact on the GI tract; therefore, studies on strain surveillance and its role in gut infection must be increased [132].

In short, an imbalance in the gut microbiota may stimulate the replication of SARS-CoV-2 and increase the production of inflammatory proteins and cytokines, leading to the development of a “cytokine storm” state. This can ultimately result in significant tissue damage, septic shock, and multiorgan failure. In summary, an imbalanced gut microbiota has the potential to facilitate SARS-CoV-2 replication and induce the release of cytokines and inflammatory proteins, ultimately leading to the onset of a severe “cytokine storm”. This, in turn, may result in significant septic shock, tissue damage, and multiorgan failure. Additional factors that can disrupt the gut microbiota in patients with COVID-19 include conditions such as diabetes mellitus, advanced age, and the administration of antibiotics, antivirals, and steroids, which can all negatively impact prognosis The severity of COVID-19 can be augmented by disruptions to the gut microbiota, which can be caused by factors such as diabetes, advanced age, and the use of medications like antibiotics, antivirals, and steroids [133].

## 10. Gut-Lung Axis in COVID-19

The interplay between the immune system’s innate and adaptive responses and the invasion of SARS-CoV-2 characterizes the onset of the COVID-19 pandemic. This interaction sets off a cascade of events that triggers inflammation and a cytokine storm in several organs, including the lungs, gut, heart, liver, and kidneys, ultimately leading to multiorgan dysfunction [134]. These pathologies overlap in respiratory and gastrointestinal tract diseases [18,19], leading to the emergence of the “gut–lung axis” concept [19]. The gut–lung axis refers to the intricate interaction between the gut and the lungs, regulated by a diverse array of immunological, neurological, and microbial mediators. The gut microbiome has emerged as a key regulator of the gut–lung axis. Specific microorganisms and their metabolites exert profound effects on pulmonary immune responses and inflammation [135]. The gut–lung axis appears to be implicated in the pathogenesis of COVID-19, as evidenced by clinical observations indicating that patients with COVID-19 who exhibit gastrointestinal symptoms are more likely to experience severe respiratory manifestations, including acute respiratory distress syndrome, hepatic injury, and shock [136,137].

The elderly are at a heightened risk of contracting SARS-CoV-2 and developing severe COVID-19, possibly due to their less diverse intestinal flora and diminished populations of beneficial microorganisms like *Bifidobacterium* spp. [17]. These findings underscore the pressing need to restore microbiota balance in COVID-19 patients. Probiotics and prebiotics have been shown to be effective in reestablishing a healthy gut/lung microbiota balance and lowering the probability of secondary infections caused by bacterial translocation [138]. Consequently, the use of probiotics and prebiotics in preventing COVID-19 and staving off disease progression to severe stages is gaining traction [139,140,141]. In the context of COVID-19, preliminary evidence suggests that the administration of probiotics and prebiotics may offer potential benefits by interacting with the lungs through the gut–lung axis or by modulating systemic inflammation. While the immunological advantages of these interventions are widely recognized, additional clinical and laboratory research is required to ascertain their specific roles in combating COVID-19 infections. Furthermore, further investigation is needed to elucidate the connection between microbial dysbiosis and COVID-19 and to determine whether dysbiosis is a cause or a consequence of the disease. Prebiotics and probiotics may help reduce the intensity and impact of the pandemic; thus, their potential usage should be investigated. 

## 11. Therapeutics

The rapid manifestation of the COVID-19 pandemic has resulted in isolation, desolation, and panic among the general masses; these have been further aggravated in the absence of any curative drugs or preventive vaccinations for this most-dreaded disease of the current century. Recognizing the severity of the current challenging situation, numerous researchers are actively exploring potential therapeutic approaches to combat the COVID-19 outbreak. This endeavor has led to the development of various drug modalities targeting COVID-19; however, definitive conclusions are still pending. Nevertheless, there is an urgent need for novel treatments to effectively address this ongoing pandemic. In-silico research has been undertaken to identify several repurposed medicines as first-line therapy for moderate-to-severe COVID-19. Some of them are antivirals (monulapiravir, favipiravir remdesivir, arbidol, baloxavir, ritonavir, lopinavir and marboxil), antiparasitic medicines (hydroxychloroquine, chloroquine, and ivermectin) and immunomodulators (tocilizumab dexamethasone, IFNs, and 2-deoxy-d-glucose) [82]. A study used ivermectin and observed that it could reduce GI complications and the number of ventilator-free days in patients with severe COVID-19 [142]. Nevertheless, further research is warranted to study the mechanism of action of ivermectin.

To treat SARS-CoV-2 infection, viral entry may be prevented by inhibiting TMPRSS2, which plays an important role in S protein priming [73]. A recent study reported that camostat (brand name: Foypan), a nonspecific serine protease inhibitor, can inhibit TMPRSS2; its use resulted in ten-fold reduced infection by SARS-CoV and SARS-CoV-2 in Calu-3 cells [73]. Selective inhibitors or RNA interference can prevent the entry of SARS-CoV-2 into cell cultures and decrease its infectivity by inhibiting the interaction between NRP1 and the S1 spike protein [73,143]. The symptomatic treatments recommended for patients with SARS-CoV-2 infection are the management of diarrhea, nausea, and vomiting. Antagonists such as a 5-hydroxytryptamine receptor, metoclopramide, and domperidone may be used for treating nausea and vomiting [38]. Considering that diarrhea is a common symptom observed in most cases of SARS-CoV-2 infection, patients are typically advised to ensure proper rehydration and monitoring of potassium levels. However, it is important to note that there is currently no evidence supporting the use of antidiarrheal drugs as a specific treatment for this symptom [4]. Although the development of diarrhea in patients with SARS-CoV-2 infection may warrant the use of antidiarrheal drugs, antimotility drugs (such as loperamide and diphenoxylate-atropine) will increase the transit time and consequently prolong the course of the infection [144]. If necessary, adsorbents (such as smectite and kaolin-pectin) can be used as antidiarrheal drugs because they bind to the digestive mucus and toxins, reducing water loss in patients with diarrhea. Antiviral drug-induced diarrhea may be treated by adjusting drug dosage because, at present, there is no drug for treating diarrhea caused by SARS-CoV-2 infection [38]. Although probiotics and dioctahedral montmorillonite can be used, which should also be considered when selecting treatments, the possibility of antibiotic-associated diarrhea or *Clostridium difficile* infection remains [38]. To develop novel treatment approaches, it is necessary to understand the immune pathways and microbiota interactions in the host and their cytokine responses [4]. Typically, managing food-borne enteric pathogens involves using active probiotic strains to promote their growth in the gut. In the context of COVID-19 infection, clinical trials have been carried out to explore the potential advantages of probiotic administration. These trials aim to investigate the efficacy of probiotics in alleviating the severity of COVID-19 symptoms and enhancing the immune system [135]. Clinical studies investigated a range of probiotic interventions, including *Lactobacillus plantarum*, *Bifidobacterium bifidum*, and a combination of *Lactobacillus acidophilus* and *Bifidobacterium lactis* [145,146,147]. The outcomes measured in these studies included changes in inflammatory markers such as C-reactive protein (CRP) and interleukin-6 (IL-6), clinical symptoms such as fever and cough, and disease severity as measured by radiological findings or hospitalization rates [145]. 

In a human clinical study, a decrease in proinflammatory biomarkers was observed in patients with colitis with both GI and non-GI conditions after 6–8 weeks of probiotic treatment [123]. A research study conducted in China examined the impact of Lactobacillus plantarum on COVID-19 patients with mild symptoms. The findings revealed that patients who received the probiotic intervention exhibited significantly reduced levels of CRP and IL-6 compared to those who received standard care without the probiotic [148]. Additionally, Zhang et al. [147] reported that a probiotic mixture containing *LactoBacillus acidophilus*, *Bifidobacterium bifidum*, and *Lactobacillus rhamnosus* improved clinical symptoms and reduced inflammatory markers in COVID-19 patients. Similarly, Gutiérrez-Castrellón et al. [149] aimed to evaluate the efficacy of a combination of four probiotic strains in COVID-19 patients who were admitted to the intensive care unit. The treatment group showed a significantly higher rate of complete remission, reduced viral load, lung infiltrates, and symptom duration, a significant increase in IgM and IgG antibodies against SARS-CoV-2, and a faster reduction in D-Dimers. These findings indicate that probiotics hold promise as a potential therapeutic approach for individuals affected by COVID-19. Although these studies provide some preliminary evidence for the potential therapeutic role of probiotics in COVID-19 management, more research is necessary to validate the beneficial effects of probiotics on COVID-19 patients.

Immunomodulatory therapies, including glucocorticoid, cytokine, and convalescent plasma therapies, are currently used to ameliorate the inflammatory status of patients with COVID-19 with gastroenteritis [37]. Certain phytocompounds in traditional medicine, including baicalin in Chinese medicine and scutellarin, hesperetin, glycyrrhizin, and nicotinamide, may be combined with ACE2 for effective treatment [37]. To prevent future CoV outbreaks, more drugs and vaccines targeting RNA viruses should be developed. At present, there is no effective targeted treatment for SARS-CoV-2 infection. Developing new therapeutic candidates that can target viral proteins, and their associated host proteins are necessary to bridge this gap, eliminate viral infection, and facilitate its clearance [82].

## 12. Limitations of the Study

Although this review provides comprehensive insights into the potential involvement of SARS-CoV-2 in gastrointestinal symptoms, it is important to acknowledge certain limitations stemming from the scarcity of information. The precise mechanisms linking gastroenteritis to the severity of COVID-19 are still not fully elucidated, and it remains unclear whether the common GI dysfunctions, including loss of appetite, anorexia, nausea, diarrhea, and vomiting, serve as protective responses against COVID-19 or are the consequences of prolonged infection with the virus. In addition, the prevalence and durability of gastroenteritis symptoms in long COVID cases are currently being examined and remain unknown, despite these symptoms being common in individuals with COVID-19. While the emerging evidence suggests a potential link between gut microbiota and COVID-19 pathogenesis, several limitations still need to be addressed. A better understanding of leaky gut associated with gut dysbiosis in COVID-19 also remains unclear. Future research should focus on elucidating the underlying mechanisms of this relationship, developing more targeted therapies, and conducting longitudinal studies to understand better the temporal relationship between changes in gut microbiota and COVID-19 severity.

## 13. Conclusions

Many patients with COVID-19 develop gastroenteritis, and many researchers have confirmed the direct infection of SARS-CoV-2 in human intestinal epithelial cells. The present review provides evidence for fecal-to-oral route transmission as a confirmation of GI infection. The presence of receptors for SARS-CoV-2 binding within the GI tract, positive results for nucleic acid testing of these viruses from intestinal samples, and occurrence of GI symptoms in approximately 25–40% of the patients prove the strong association between CoVs and GI tract infections. To control the symptom of diarrhea, it is necessary to first identify its cause (whether it is due to SARS-CoV-2 infection or the use of other therapeutics such as antiviral agents, antibiotics, and certain traditional Chinese medicines). Patients exhibiting GI manifestations should receive treatments aimed at preserving GI functions. Additionally, further research is needed to explore the alterations in the gut microbiota caused by SARS-CoV-2 infection, with the goal of developing effective strategies to leverage these changes for gastroenteritis treatment. Gastroenterologists should also prioritize vigilance toward atypical digestive symptoms in infected individuals to prevent viral transmission through various potential routes and effectively manage the spread of COVID-19. Future research should be focused on developing SARS-CoV-2 resistance in patients by modulating the gut microflora, using prebiotics and probiotics (mainly to understand the GI tract’s bacterial dynamics), and using the novel approach of fecal transplant. Furthermore, drugs and vaccines targeting multiple viral RNAs should be developed to control CoVs. Studies on the host cell’s immune response to viral infection are also warranted to understand the virulence of these viruses and to develop more effective drugs capable of promoting the immune system. Individuals with deficient HD5, as well as those with IBDs such as UC and CD, or individuals who have undergone small intestine transplantation, are more vulnerable to SARS-CoV-2 infection compared to healthy individuals. Exploring the mechanisms of SARS-CoV-2 entry into the intestinal tract, the resulting gastrointestinal symptoms, and the host’s immune response to the infection could aid in the development of improved therapeutic strategies for managing viral infections in patients. Understanding the gastrointestinal manifestations of COVID-19 is important for several reasons. First, it can help healthcare providers recognize and manage GI symptoms in COVID-19 patients, which can improve patient outcomes. Second, it may help identify potential transmission routes for the virus, which can inform public health interventions. Finally, investigating the effects of COVID-19 on the GI tract could lead to the development of novel treatment approaches for COVID-19 patients experiencing GI complications.

## Data Availability

No new data created.
